# A Rare Presentation of Ganglion Cyst of the Elbow

**DOI:** 10.7759/cureus.665

**Published:** 2016-07-01

**Authors:** Raju Vaishya, Chirag Kapoor, Amit Kumar Agarwal, Vipul Vijay

**Affiliations:** 1 Orthopaedics, Indraprastha Apollo Hospitals; 2 Orthopaedics, Sumandeep Vidyapeeth, Vadodara, Gujarat

**Keywords:** ganglion, cyst, elbow, diagnosis

## Abstract

INTRODUCTION: Ganglion cysts are benign soft tissue swellings commonly found in the wrist. The presence of these cysts in the elbow is uncommon, and few case reports have been reported for this condition at this location. These lesions can compress on the neighbouring structures or cause restriction of the joint movement. The awareness of this entity is a must, to arrive at an early diagnosis.

MATERIALS AND METHOD: We report a patient with swelling in the anterolateral aspect of the elbow which had been causing intermittent pain for the last 13 months. The MRI revealed a fluid-filled cystic swelling which was communicating with the radio-capitellar joint.

RESULTS: The lesion was excised in toto, using anterolateral approach for the elbow, and sent for histopathological examination which confirmed the diagnosis of a ganglion cyst.

CONCLUSION: Thus, due to the infrequent presentation, an awareness of this condition is necessary to prevent a delay in diagnosis and its subsequent management.

## Introduction

Ganglion cysts are the most common benign soft tissue swellings, which can be found in any joint of the body, but 60-70% are found in the dorsal aspect of the wrist and communicate with the joint via a pedicle [1]. Occurrence of ganglion cysts in other joints such as the elbow is rare. These are well defined and mobile swellings, which are loosely attached to the sheath of the tendon or the capsule of the joint.

Ganglion cysts around the elbow are rare [2]. Hence, the diagnosis of these lesions may be challenging and often delayed if the awareness of this entity is not known to the clinician. These are usually asymptomatic but sometimes may cause restriction of elbow range of movement or compression of radial or posterior interosseous nerve. Most of these cysts are seen as an incidental finding on Magnetic Resonance Images (MRI) or on ultrasonography (USG). We report the case of a ganglion cyst in the elbow (originating from the radio-capitellar joint), in a middle-aged female where the diagnosis could not be made for more than a year.

## Case presentation

A 45-year-old woman presented with a 13-month-history of pain and swelling in the anterior aspect of the right elbow. There was no history of trauma or any other exacerbating factor. She had been taking analgesics for last nine months due to intermittent pain which affected her ability to use the dominant elbow.

Local examination revealed a 3 cm x 3 cm soft tissue swelling on the anterolateral aspect of the elbow, which was tender on palpation. The swelling was mobile in all directions. Elbow range of movement was from zero degrees to 125 degrees. There was no associated erythema around the elbow and over the swelling.

**Figure 1:**
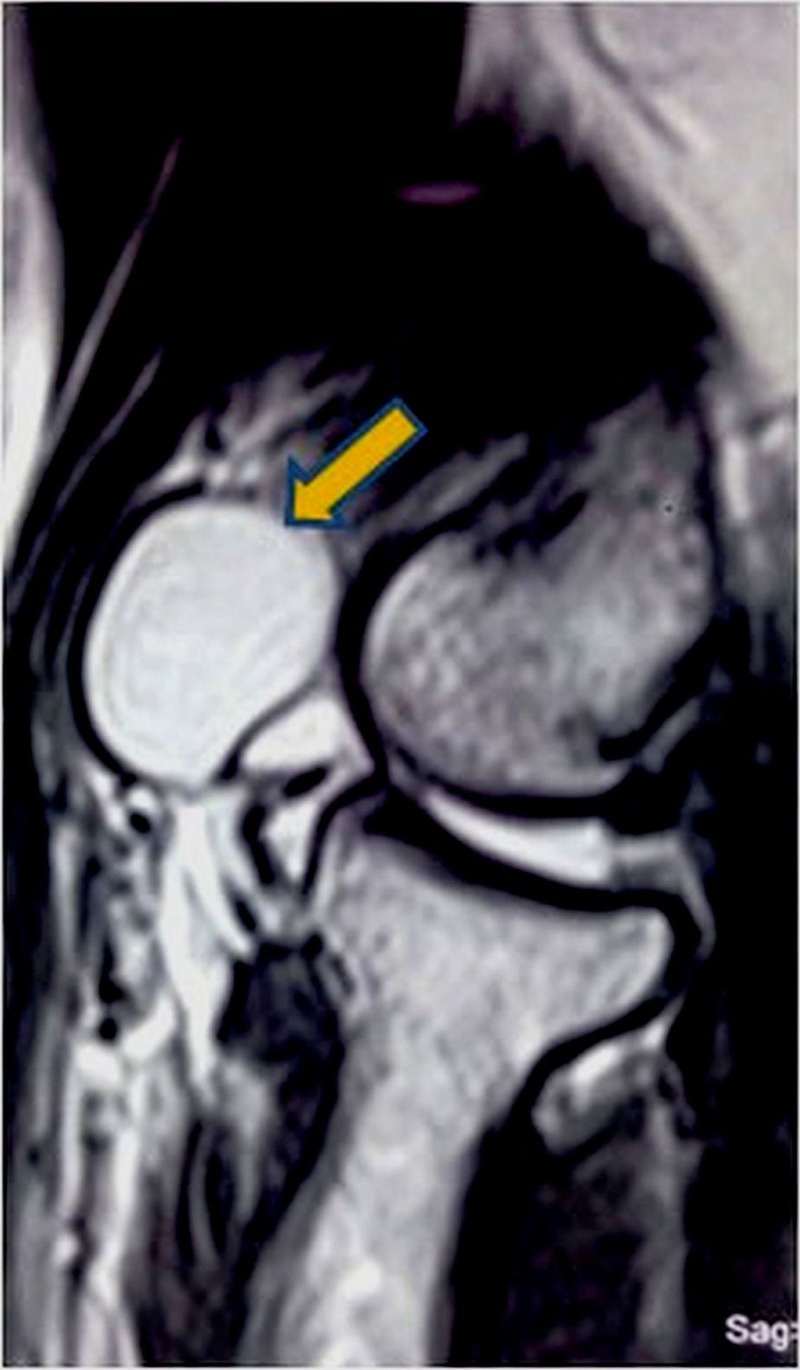
MRI T2 weighted saggital view showing hyerintense cyst anterior to radiocapitellar joint

**Figure 2:**
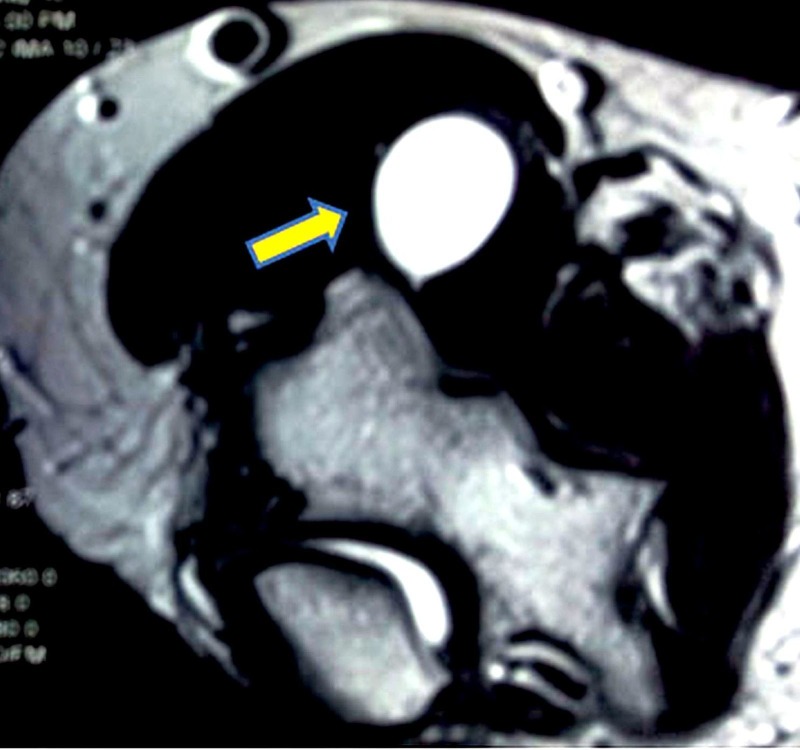
MRI (T2 weighted) cross section view showing the cyst in the anterolateral aspect of the elbow

The MRI showed a multiseptate fluid collection measuring 1.73 x 1.32 cm on the lateral aspect of cubital fossa anterior to the radio-capitellar joint space possibly communicating with it showing T1 hypo and T2/STIR hyperintense signals. Anteriorly it was burrowed into the brachioradialis muscle (Figure 1, 2).

**Figure 3:**
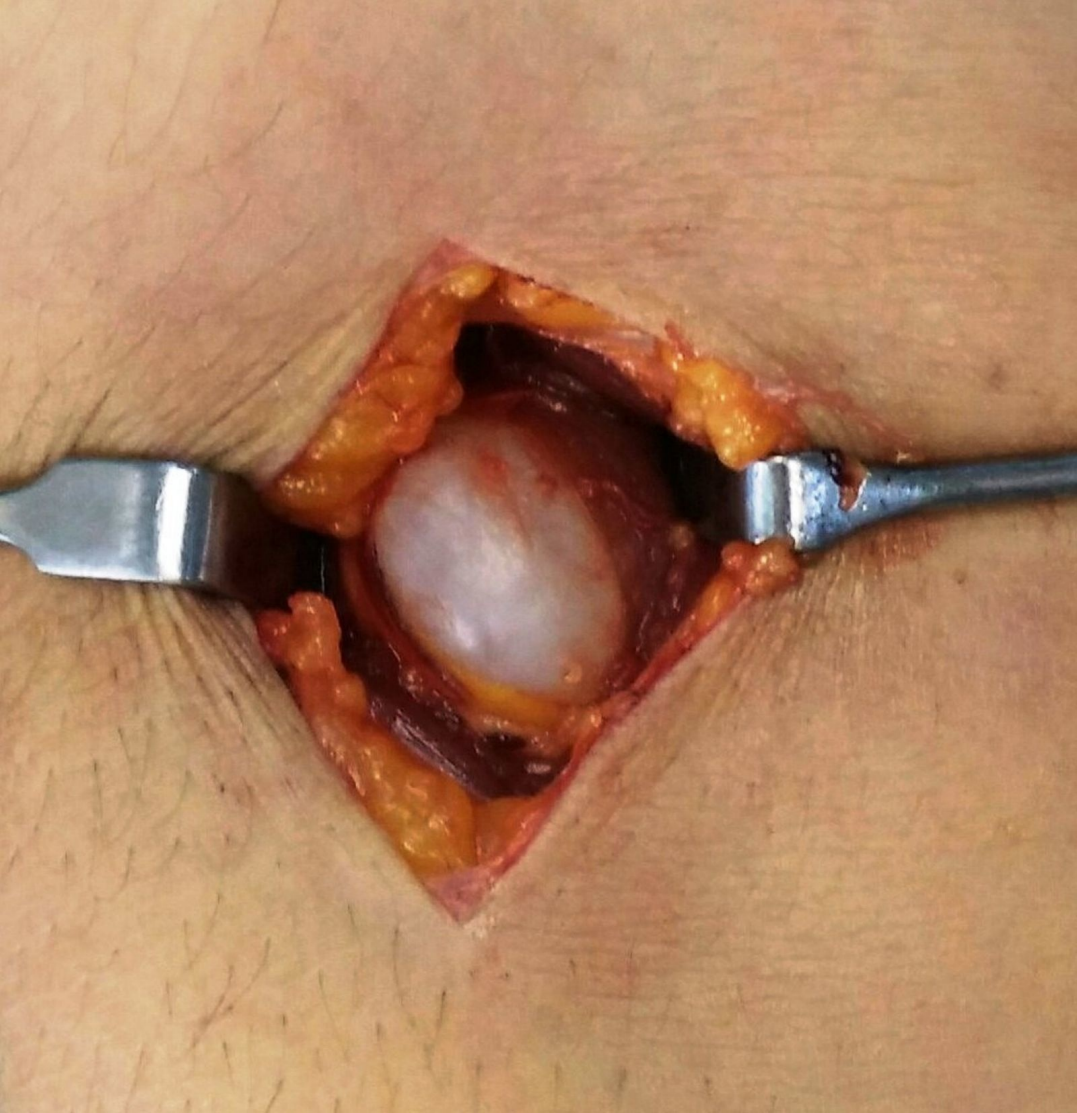
Intraoperative picture showing the cyst

This symptomatic swelling was excised through an anterolateral approach to the elbow joint [3], after making a plane between brachialis and brachioradialis. A cystic swelling of about 2 cm x 2 cm in size was found (Figure 3).

**Figure 4:**
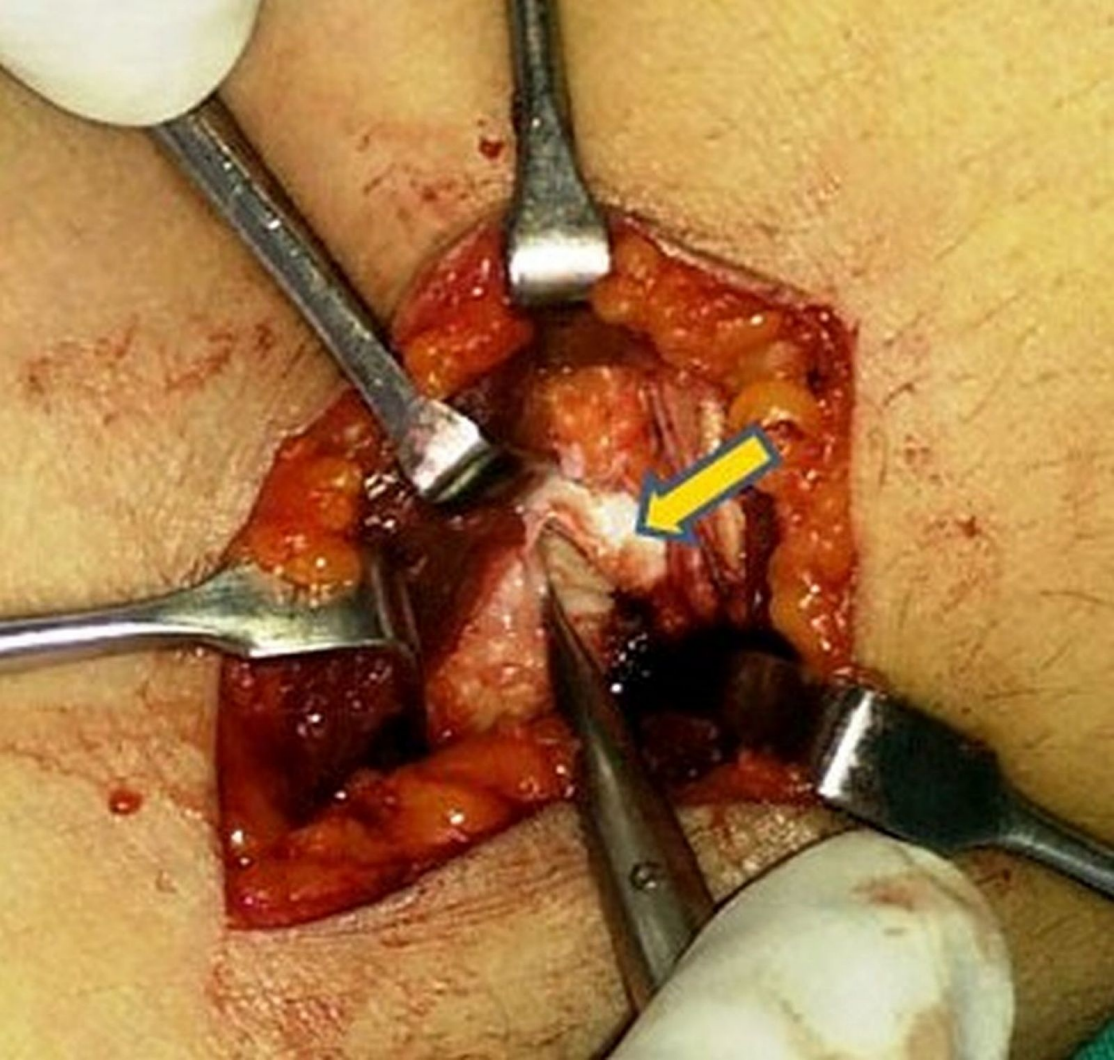
Intraoperative picture showing rent in the capsule of radiocapitellar joint

It was carefully isolated from the surrounding soft tissues and excised en-masse. The stalk of the lesion was found to be originating from the radio-capitellar joint (Figure 4).

**Figure 5:**
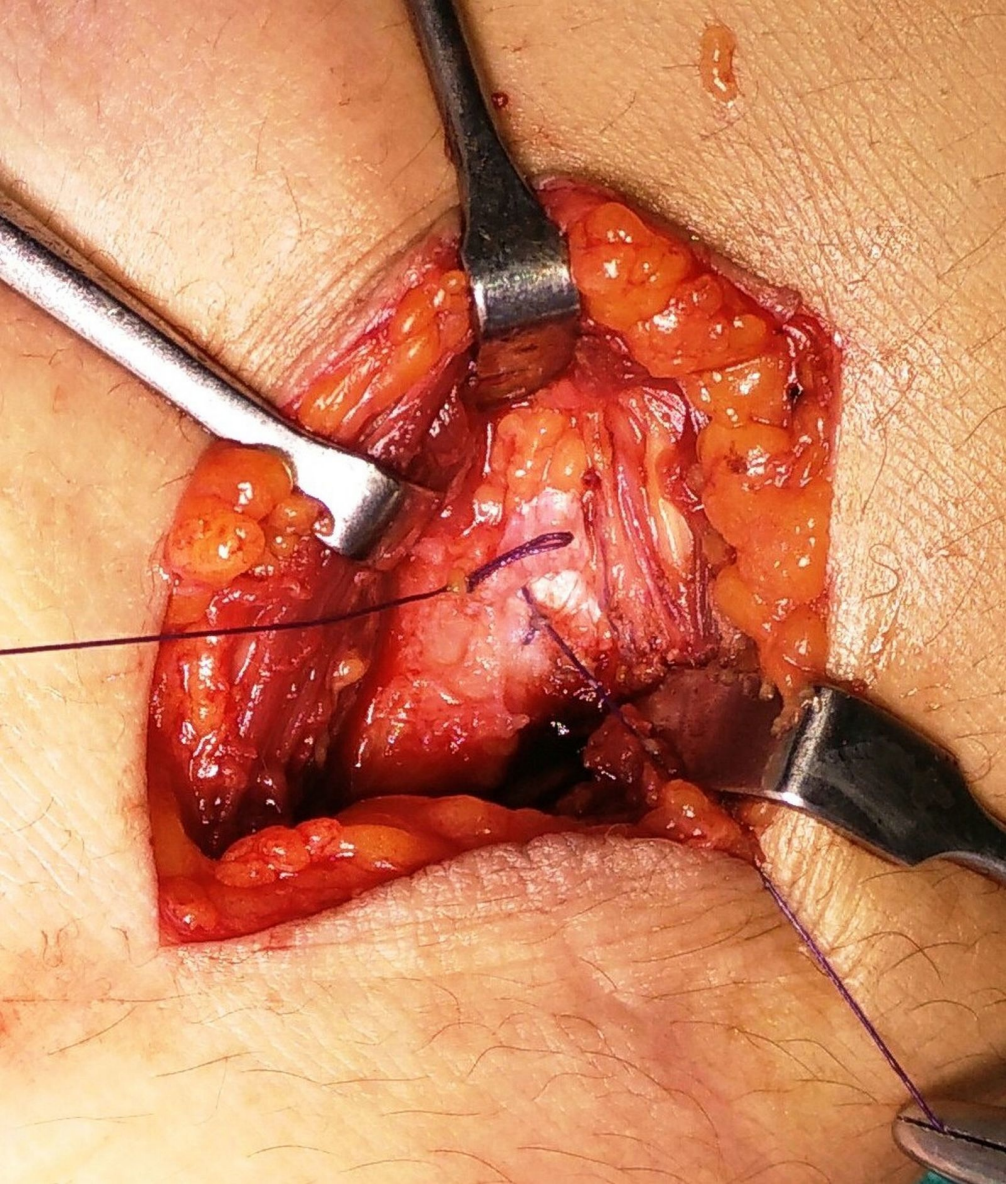
Intraoperative picture showing repair of the capsule after excision of the cyst

The rent in the capsule was then closed with an absorbable Vicryl 3-0 suture (Figure 5).

The excised lesion was sent for histopathological examination, which confirmed the diagnosis of a ganglion cyst.

Written consent was obtained from the human subject who participated in this case study. Institutional Review Board of Indraprastha Apollo Hospital, India approved the research for this case.

## Discussion

The clinical diagnosis of an elbow swelling is often challenging, due to the complex nature of the joint and rarity of the location of the swelling. Such lesions may be a manifestation of a wide variety of conditions like myositis ossificans, ganglion cysts, lipoma, cysticercus cyst, vascular aneurysms, soft tissue sarcoma, septic arthritis, lymphoedema, and pseudogout.

A confirmatory diagnosis can be made using plain radiographs, USG and MRI [4]. We believe that an MRI is the investigation of choice for soft tissue lesions, as one can assess its site of origin, the size of swelling and whether it is compressing on any nerve or vessel around the elbow. Ganglion cyst on MRI has classical features viz. unilocular or multilocular rounded or lobular fluid signal mass (which is hyperintense on T2/STIR and hypointense signals on T1 sequences), adjacent to a joint or tendon which distinguishes it from the other pathologies.

Treatment modalities of ganglion cysts include conservative management by aspiration, injection of steroids, sclerosing agents and hyaluronidase and thread technique. However, most of these modalities are associated with either high recurrence rates or other complications like incomplete resolution and allergic reactions [5]. Steroid therapy is associated with subcutaneous fat atrophy and depigmentation of the skin [6]. Sclerotherapy is known to cause damage to the tendon from which the ganglion cyst arises [7]. The most efficient modality for treating the ganglion is to excise the cyst in toto and repair the rent in the capsule or tendon sheath, which would reduce the rate of recurrence of the tumor [8]. The recurrence rate after excision of the lesion, in current literature, is variable and ranges from 0 - 31.2 %.

There are a few reported cases of ganglion cyst at the elbow, and most of them have been shown to cause compressive neuropathies of the radial nerve or the posterior interosseous nerve [9]. A ganglion cyst in the supinator muscle was reported, which caused compression of the posterior interosseous nerve leading to weakness of wrist extensors [10]. Matsubara et al. reported 8 cases of radial nerve palsy due to ganglions at the elbow, with majority of cases presenting no symptoms [11]. We did not find any evidence of any compressive neuropathy in our case. 

## Conclusions

Ganglion cysts are common, benign, soft tissue swellings found around the joints. Their presence in the elbow is uncommon, and only few case reports have been noted for this condition at this location. Due to an uncommon presentation in the elbow, the ganglion cysts are often difficult to diagnose. Hence, a delay in diagnosis is inevitable, just as it occured in our case. Hence, an awareness about the rare presentation of a ganglion cyst in the elbow is necessary, so as to arrive at an early diagnosis and treatment.
